# Utilization of the Lempert Maneuver for Benign Paroxysmal Positional Vertigo in the Emergency Department

**DOI:** 10.7759/cureus.24288

**Published:** 2022-04-19

**Authors:** Vanessa Hwu, Arielle K Burris, Jessica R Pavolko, Daniel T Sawyer, Marna R Greenberg, David B Burmeister

**Affiliations:** 1 Department of Emergency and Hospital Medicine, University of South Florida (USF) Morsani College of Medicine/Lehigh Valley Health Network, Bethlehem, USA; 2 Department of Physical Therapy and Occupational Therapy, University of South Florida (USF) Morsani College of Medicine/Lehigh Valley Health Network, Allentown, USA

**Keywords:** benign paroxysmal positional vertigo, physical therapy, lempert maneuver, canalithiasis, vertigo

## Abstract

Benign paroxysmal positional vertigo (BPPV) is a common cause of vertigo. Symptoms from BPPV lead to Emergency Department (ED) visits, and persistence of symptoms, particularly in the elderly, may impact patient disposition. We describe the techniques used in the case of a 72-year-old male with dizziness, who had symptom resolution, and was able to be safely discharged after a Lempert maneuver (barbeque (BBQ) roll) was performed in the ED setting. The patient presented to the ED with left gaze nystagmus, and otherwise normal evaluation results. Physical therapy was consulted, and their initial evaluation indicated right horizontal canalithiasis noted by fatiguing right, geotropic nystagmus, but the patient was unable to tolerate further testing due to vomiting. Antiemetic medications were administered and at his follow-up examination an hour later, a total of three Lempert maneuvers were performed, resulting in total symptom resolution. Successful utilization of the Lempert maneuver to treat BPPV can help to reduce ED length of stay and increase patient satisfaction. Because of this, the Lempert maneuver should be considered a fast, cost-effective, and safe method of alleviating BPPV symptoms.

## Introduction

Benign paroxysmal positional vertigo (BPPV) is a common cause of vertigo and is seen in up to 50% of elderly patients who present with dizziness [1]. BPPV occurs when calcium debris is found in the semicircular canals (canalithiasis) of the inner ear causing endolymph in the canals to move inappropriately [2]. Patients often present with recurrent episodes of vertigo that are commonly triggered by head movements such as looking up, lying down, or rolling over. Nausea and vomiting may accompany these episodes, but other neurologic complaints are typically absent. Differentiating between the types of BPPV, posterior canal and horizontal canal, is done by observing nystagmus during provoking maneuvers such as the Dix-Hallpike maneuver for posterior canal BPPV [3] or the head-roll test for horizontal canal BPPV [4].

Depending on the type of BPPV, different repositioning maneuvers can be used to encourage the calcium debris back into the utricular sac [3]. One such maneuver, known as the Lempert maneuver, Lempert 360 roll, log roll, or barbeque (BBQ) roll, involves having the individual remain in the supine position with their cervical spine flexed to about 30 degrees with their head in a neutral, centered position then rotating the individual's head 90 degrees toward the affected side with each change in head position being held for 30 seconds after the nystagmus and vertigo symptoms stop [5]. The head is then rotated back to neutral, then 90 degrees away from the affected side, then into a prone position and if nystagmus and vertigo symptoms persist, multiple BBQ rolls may be required [5].

Physical therapy treatment of BPPV is highly effective, can provide rapid symptom relief [3], and prevents unnecessary testing [5]. In addition to these benefits, it can be performed either by physical therapists or by the emergency physician themselves with little to no physical risk to the patient. Outpatient instructions can even be provided to family members to perform the maneuver in the comfort of their own home, although training should be provided to family members in this case, as untrained persons can have a large margin of error when performing the BBQ roll at home [6]. We present a case in which a patient with BPPV was successfully treated with a BBQ roll and able to be discharged from the Emergency Department (ED).

## Case presentation

A 72-year-old male with a history of vertigo, atherosclerosis, coronary artery disease, and hypertension presented to the ED on Christmas weekend, during the coronavirus disease 2019 (COVID-19) pandemic, with a complaint of dizziness. He had no related surgery or trauma and three months prior he had an incidental bilateral L 4/5 transforaminal steroid injection procedure. He noted that he has had dizziness for the past year since having COVID-19, but his symptoms have progressively worsened and were exacerbated by positional changes. At the time of this encounter, he was taking 300 mg aliskiren, 200 mg allopurinol, 10 mg atorvastatin, 10 mg nebivolol, 20 mg paroxetine, and 0.4 mg tamsulosin daily. Though aliskiren, nebivolol, paroxetine, and tamsulosin can all have side effects that include dizziness, none are associated with persistent, positional vertigo. He endorsed symptoms of nausea and vomiting but denied any neurologic or cardiac complaints. On examination, he was afebrile, his blood pressure was 160/100, heart rate was 94, and respiratory rate was 20 with an oxygen saturation of 95% on room air. A left gaze nystagmus was noted but the rest of his findings were within normal limits. A CT head without contrast was ordered, which showed no acute changes. Physical therapy (PT) was consulted for symptomatic treatment of BPPV.

**Figure 1:**
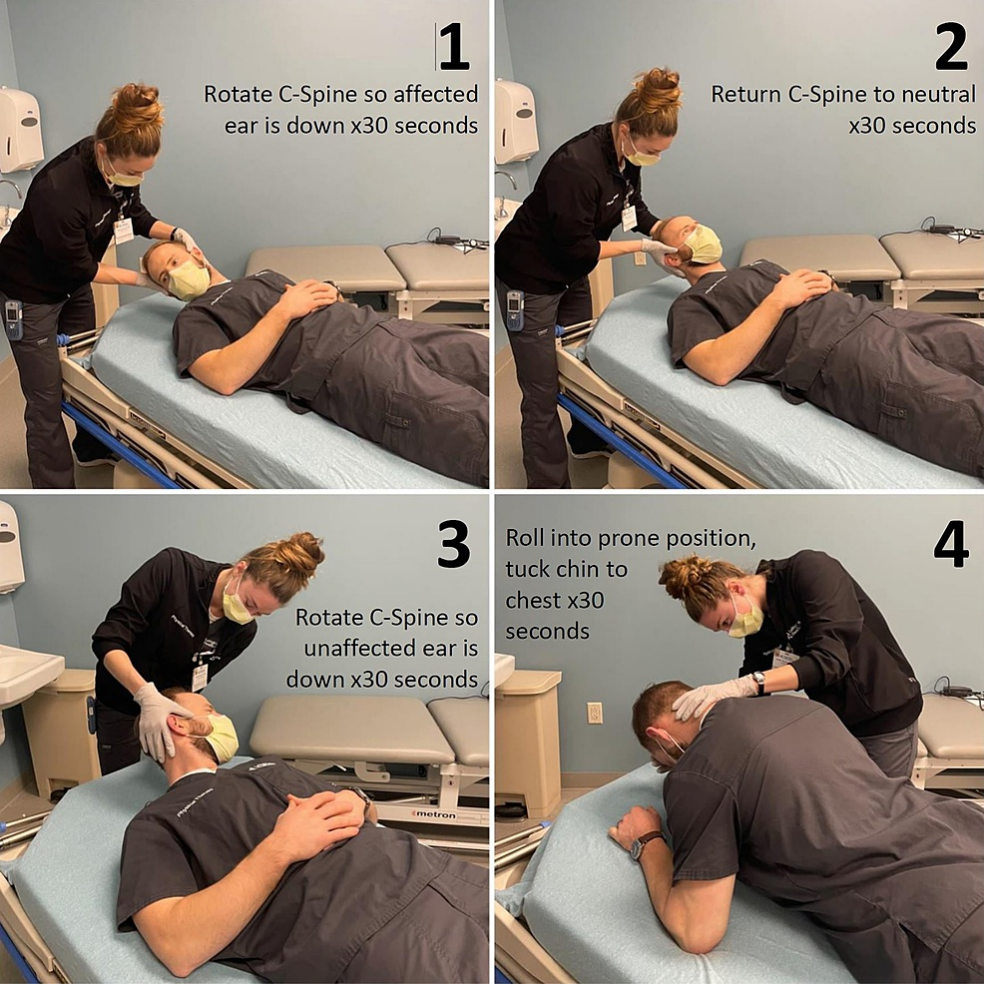
An explanation of the application of the Lempert (BBQ roll) maneuver. Panels 1-4 highlight the individual steps of the procedure. The subjects shown are authors Arielle Burris and Daniel Sawyer, Physical Therapists BBQ: barbeque

Initial evaluation by PT indicated right horizontal canalithiasis noted by fatiguing right, geotropic nystagmus on right roll test. The patient was unable to tolerate further vestibular testing due to vomiting, and antiemetics were administered. At this time, he did not meet criteria for discharge from a functional standpoint. At his follow-up evaluation an hour later, he was able to tolerate three BBQ rolls, or Lempert maneuvers (Illustrated in Figure 1 and Video 1). After the third roll, he no longer exhibited nystagmus and had no complaints of room spinning. He then ambulated 61 meters with a steady gait. He was deemed to be fit for discharge and he and his wife were educated on outpatient PT follow-up.

**Video 1:** A demonstration of the Lempert (BBQ roll) maneuver. The subjects in the video are authors Arielle Burris and Daniel Sawyer, Physical Therapists BBQ: barbeque

## Discussion

Upon initial evaluation, amidst a crowded waiting room during a holiday weekend COVID-19 surge, and with no readily available observation or inpatient beds, the patient did not meet functionality criteria for discharge due to persistent BPPV symptoms. The availability of physical therapy in the ED helped mitigate overcrowding and provide a higher quality of care by quickly stabilizing an elderly man for discharge.

In the wake of the COVID-19 pandemic, some evidence has emerged that indicates an increased incidence of both types of BPPV among those who were previously infected with and hospitalized for COVID-19 [7]. These data would indicate that EDs may see an increased number of BPPV-related cases. This further increases the importance of developing a rapid and effective method of ameliorating BPPV.

In the context of the COVID-19 pandemic, early interdepartmental resource utilization, when they are institutionally available, is vital to the quality management of patients, as well as overall management of high-volume demand. There seems to be a resistance to provider performance of these maneuvers. If a clinician cannot perform the maneuver, consulting someone who can is beneficial to the patient. Having PT in the ED has also been shown to decrease wait times and lower burden on ED physicians [8]. During this time when hospitals are reaching maximum capacity, utilizing every resource available to limit the number of admissions and prevent ED overcrowding is key in providing quality care [9]. Additionally, avoiding hospital admission benefits the patient by decreasing the risks associated with hospitalization such as nosocomial infections, functional decline, delirium, and polypharmacy [10]. Having an interdisciplinary team available in the ED helps with patient satisfaction, quality of care, and hospital operation.

## Conclusions

Utilization of the Lempert (BBQ roll) maneuver can effectively alleviate symptomology from BBPV. Due to its ease of use and rapid symptom resolution, the Lempert maneuver can help to alleviate ED overcrowding by decreasing ED length of stay in BPPV patients. As such, it should be considered in the approach to patients with BPPV in the ED setting. In addition to these benefits, providing patients with a more rapid symptom resolution to an extremely disconcerting condition such as BPPV helps to improve the patient experience and quality of care that they receive.
